# Toward standardized continuous glucose monitoring validation: lessons from the Sinocare iCan i3 accuracy study

**DOI:** 10.3389/fcdhc.2026.1941126

**Published:** 2026-09-16

**Authors:** Auda Ameur

**Affiliations:** 1 Department of Pediatrics B ‘Amilcar Cabral, University Hospital of Oran, Oran, Algeria; 2 Faculty of Medicine, University of Oran 1, Oran, Algeria

**Keywords:** accuracy, blood glucose monitoring, continuous glucose monitoring, diabetes, MARD, methodology, real-world evidence, sensor validation

## Abstract

Continuous glucose monitoring (CGM) has transformed diabetes management by providing real-time glucose measurements and supporting improved glycemic control. Consequently, the methodological quality of CGM accuracy studies has become increasingly important for clinicians, regulatory agencies, and healthcare decision-makers. The recent real-world evaluation of the Sinocare iCan i3 CGM system provides valuable evidence regarding device performance under routine clinical conditions. However, several methodological aspects warrant further consideration before the reported findings can be generalized to clinical practice. This perspective critically examines key aspects of CGM validation, including study design, selection of the reference glucose measurement, statistical methodology, temporal pairing strategies, sensor survival analysis, potential sponsorship-related bias, and interpretation of clinical accuracy metrics. We also discuss additional methodological considerations that were not fully addressed in the original publication, including uncertainty estimation, subgroup analyses, longitudinal sensor performance, missing-data mechanisms, and reproducibility assessment. Finally, we propose recommendations aimed at improving the methodological rigor of future real-world CGM validation studies and facilitating meaningful comparisons across CGM technologies.

## 1 Introduction

Continuous glucose monitoring (CGM) technologies have evolved considerably over the past decade, with modern systems achieving substantial improvements in analytical performance and widespread clinical adoption (1, 2). Beyond glucose monitoring itself, CGM has become an integral component of diabetes management, supporting therapeutic decision-making, hypoglycemia prevention, and optimization of long-term glycemic control (3, 4). Consequently, the analytical validity of CGM accuracy studies has important implications for clinical decision-making, patient safety, regulatory evaluation, and confidence in emerging technologies (5).

Despite these technological advances, the methodological frameworks used to evaluate CGM performance have evolved less consistently. Most validation studies continue to rely primarily on global mean absolute relative difference (MARD), error grid analyses, and comparisons with laboratory or capillary reference glucose measurements. Although these approaches remain valuable, no single metric can fully capture the multidimensional performance of contemporary CGM systems. Moreover, substantial heterogeneity in reference standards, participant selection, glucose-pairing strategies, statistical analyses, reporting practices, and assessment of clinical outcomes continues to limit meaningful comparisons across devices and complicate interpretation of the published evidence.

The recent real-world evaluation of the Sinocare iCan i3 CGM system provides an illustrative example of these broader methodological challenges. Rather than focusing solely on the performance of a single device, its evaluation provides an opportunity to examine issues relevant to contemporary CGM validation, including the selection of reference methods, physiological delays between blood and interstitial glucose, characterization of statistical uncertainty, longitudinal assessment of sensor performance, evaluation of missing data, and methodological standardization. As analytical differences among current-generation CGM systems become progressively smaller, variability in study methodology may increasingly influence reported accuracy estimates and complicate direct comparisons among devices.

The next major advance in diabetes technology may therefore depend not only on improvements in sensor chemistry and signal-processing algorithms but also on the development of more harmonized approaches to CGM validation. Methodological standardization has improved the quality and transparency of other areas of clinical and diagnostic research through initiatives such as STARD, CONSORT, and TRIPOD. A comparable framework specifically adapted to CGM evaluation could enhance reproducibility, facilitate more objective comparisons among devices, and strengthen the evidence supporting clinical and regulatory decision-making.

Accordingly, the objective of this Perspective is not to criticize a single study, but to use the Sinocare iCan i3 evaluation as an illustrative case through which broader methodological issues in CGM validation can be examined. We propose a multidimensional framework emphasizing standardized reference methodology, complementary analytical and clinical performance metrics, longitudinal assessment of sensor accuracy, robust statistical approaches, transparent reporting, and methodological harmonization. As next-generation CGM technologies continue to evolve, their evaluation methods should advance with comparable rigor to ensure that improvements in analytical performance are supported by equally robust scientific evidence.

## 2 Methodological approach

This Perspective is based on a narrative critical appraisal of the recent real-world evaluation of the Sinocare iCan i3 continuous glucose monitoring (CGM) system, which is used as an illustrative case study to examine broader methodological challenges in CGM validation. The appraisal was informed by current international clinical practice guidelines, methodological recommendations, and recent literature on CGM performance evaluation, with particular attention to reference glucose measurement, accuracy metrics, statistical methodology, longitudinal performance assessment, and reporting transparency. Rather than constituting a systematic review, this Perspective aims to synthesize relevant evidence and identify methodological considerations that may improve the design, analysis, interpretation, and comparability of future real-world CGM validation studies.

The principal methodological recommendations derived from this appraisal are summarized in **Table 1**. They are intended to promote greater methodological consistency across future CGM validation studies, facilitate meaningful comparisons among devices, and strengthen their analytical, clinical, and regulatory evaluation.

**Table 1:** Proposed best practice recommendations for standardized CGM validation studies.

Methodological domain	Current practice in many CGM validation studies	Recommended best practice
Reference glucose measurement	Capillary blood glucose meter (BGM) used as the reference	Prefer laboratory YSI plasma glucose measurement whenever feasible; if BGM is used, report analytical bias, precision, and reference measurement uncertainty
Accuracy metrics	Reliance on overall MARD alone	Report overall and glucose-range-specific MARD together with MedARD, MAD, RMSE, Bland–Altman analysis, and systematic bias
Clinical accuracy	Clarke Error Grid only	Include Clarke, Parkes (Consensus), and Surveillance Error Grid analyses, together with assessment of clinical decision impact
Reference pairing strategy	Single fixed matching window (± 5 min)	Perform sensitivity analyses using ±1-, ± 2-, and ±5-minute windows and stratify analyses according to glucose rate of change
Longitudinal performance	Overall pooled accuracy	Evaluate daily MARD, sensor ageing, temporal drift, and end-of-wear performance
Missing data	Rarely reported or excluded	Report missing-data mechanisms (MCAR, MAR, MNAR) and perform multiple imputation and sensitivity analyses when appropriate
Statistical uncertainty	Point estimates only	Report 95% confidence intervals and bootstrap-derived uncertainty estimates for primary accuracy outcomes
Sensor survival	Descriptive reporting	Use Kaplan–Meier survival analysis and Cox proportional hazards regression, when appropriate, to investigate predictors of premature sensor failure
Subgroup analyses	Limited or absent	Perform predefined subgroup analyses according to relevant characteristics, such as diabetes type, age, BMI, HbA1c, insulin therapy, pregnancy, renal impairment, and ethnicity
Reproducibility	Single-sensor evaluation	Assess intra-sensor and inter-sensor reproducibility using repeated sensor insertions whenever feasible
Transparency and reproducibility	Basic methodological reporting	Preregister study protocols, publish statistical analysis plans, ensure independent statistical review, transparently disclose funding, and encourage sharing of anonymized paired data

BGM, blood glucose monitoring; BMI, body mass index; CGM, continuous glucose monitoring; MAD, mean absolute difference; MAR, missing at random; MCAR, missing completely at random; MedARD, median absolute relative difference; MNAR, missing not at random; MARD, mean absolute relative difference; RMSE, root mean square error; YSI, Yellow Springs Instrument.

These recommendations are intended to improve methodological rigor, facilitate meaningful comparisons among CGM validation studies, and strengthen the analytical, clinical, and regulatory evaluation of current and future CGM systems.

## 3 Methodological appraisal of contemporary CGM validation

This Perspective builds on methodological considerations previously raised regarding the real-world evaluation of the Sinocare iCan i3 CGM system by examining additional factors that may influence the interpretation of analytical accuracy. Although the study provides valuable real-world evidence, several methodological choices warrant further consideration when interpreting the findings and assessing their generalizability to clinical practice (6–8).

### 3.1 Reference standard selection

A key methodological consideration concerns the selection of the reference glucose measurement. The investigators used capillary glucose measurements obtained with the FreeStyle Optium system rather than a laboratory reference method such as the Yellow Springs Instrument (YSI) (6). Although this approach reflects routine clinical practice and facilitates home-based evaluation, it introduces additional analytical uncertainty because blood glucose meters have their own measurement error, bias, and imprecision.

Consequently, the reported MARD reflects not only CGM performance but also the combined measurement variability arising from both the CGM system and the reference method. This may lead to either overestimation or underestimation of the sensor’s analytical performance. Future validation studies using capillary glucose measurements as the reference should therefore characterize the analytical uncertainty of the reference method and, where feasible, consider its contribution when interpreting MARD estimates.

### 3.2 Interpretation of MARD

MARD remains the most frequently reported indicator of CGM accuracy (14). Nevertheless, increasing evidence suggests that MARD alone is insufficient for comprehensive device evaluation. MARD is highly dependent on glucose distribution, sample composition, glycemic variability and study design. Devices with similar MARD values may demonstrate markedly different clinical performance (14).

The study mainly emphasizes the overall MARD of 17.2% (6). However, additional performance indices, including Median Absolute Relative Difference (MedARD), Mean Absolute Difference (MAD) during hypoglycemia, root mean square error (RMSE), and distribution of absolute relative differences across glucose ranges, would provide a more comprehensive description of analytical behavior.

### 3.3 Quantitative interpretation of reported accuracy estimates

Beyond the methodological considerations discussed above, the numerical performance estimates reported in the original study also warrant careful interpretation. The real-world evaluation included 36 participants with type 1 or type 2 diabetes who wore the Sinocare iCan i3 CGM system for 15 consecutive days, yielding an overall MARD of 17.2% (6). Furthermore, 69.5% of paired CGM-reference measurements met the predefined agreement criterion of ±20 mg/dL or ±20%, while 77.1% of sensors remained functional through the end of the wear period (6).

Although these findings provide an initial overview of analytical performance, they should be interpreted within the context of the study methodology. The reported MARD of 17.2% is higher than values reported for several established contemporary CGM systems evaluated using laboratory reference methods (11–14). However, direct numerical comparisons should be made cautiously because differences in reference methods, study populations, glucose distributions, pairing strategies, and study settings may substantially influence reported accuracy estimates. Accordingly, the observed difference may reflect not only sensor characteristics but also methodological differences between validation studies. Similarly, the finding that 69.5% of paired observations met the ±20/20 agreement criterion indicates that approximately 30% fell outside this predefined threshold, supporting the importance of examining error distributions across glucose ranges rather than relying exclusively on a single summary measure.

The reported 77.1% sensor survival rate also indicates that approximately one-quarter of sensors did not complete the intended 15-day wear period (6). Beyond its implications for device durability, this attrition raises the possibility of survival bias if sensors that were discontinued prematurely differed systematically in analytical performance from those that completed the study period. Without a comparative analysis of performance according to sensor completion status, the potential magnitude and direction of this bias cannot be determined. Therefore, pooled accuracy estimates should be interpreted alongside information on sensor discontinuation and longitudinal performance.

Taken together, these quantitative findings support the value of complementing summary accuracy indices with measures of statistical uncertainty, longitudinal performance analyses, subgroup-specific evaluations, and sensitivity analyses to provide a more comprehensive assessment of real-world CGM performance (9).

For contextual interpretation, **Table 2** summarizes the principal methodological characteristics and reported analytical performance of selected contemporary CGM systems (10–14). These comparisons are intended to illustrate methodological heterogeneity rather than establish a definitive ranking of device performance. Differences in reported accuracy should therefore be interpreted in the context of variations in reference methods, study design, participant characteristics, wear duration, and analytical approaches (9–14).

**Table 2:** Comparative methodological characteristics and analytical performance of selected contemporary continuous glucose monitoring systems.

Device	Study (year)	Reference method	Sample size	Wear duration	Overall MARD (%)	Clinical accuracy	Main strengths	Main methodological limitations	Potential sources of methodological heterogeneity
Sinocare iCan i3	Bhargava et al. (2025) (6)	FreeStyle Optium capillary BGM	36	15 days	17.2	98.8% Clarke Error Grid Zones A+B	Real-world home-use evaluation; factory-calibrated system; assessment throughout full sensor wear	Capillary BGM rather than YSI laboratory reference; small sample size; ± 5-minute pairing window; no longitudinal accuracy analysis; sensor survival of 77.1%; limited uncertainty analysis	Capillary reference method, small single-center cohort, real-world home conditions, and differences in analytical methodology
Dexcom G7	Garg et al.	YSI laboratory reference	>300	10 days	~8.2	>99% Consensus Error Grid Zones A+B	Large multicenter study; YSI reference; comprehensive statistical evaluation	Conducted mainly under controlled clinical conditions, with limited real-world assessment	YSI-based reference assessment, large multicenter cohort, standardized analytical methods, and predominantly controlled study conditions
FreeStyle Libre 3	Christiansen et al.	YSI laboratory reference	>100	14 days	~7.8	>99% Consensus Error Grid Zones A+B	High reported analytical accuracy; assessment of hypoglycemic-range performance; multicenter validation	Limited evaluation of long-term real-world adherence and routine home use	YSI-based assessment, multicenter design, standardized laboratory procedures, and predominantly controlled validation conditions
Medtronic Simplera™	Carlson et al.	YSI laboratory reference	>100	7 days	~8.7	>99% Consensus Error Grid Zones A+B	Calibration-free design; multicenter validation; robust reported analytical performance	Limited post-marketing and real-world performance data	YSI-based evaluation, multicenter clinical design, standardized analytical procedures, and limited real-world assessment
Dexcom G6	Wadwa et al.	YSI laboratory reference	>300	10 days	~9.0	>99% Consensus Error Grid Zones A+B	Extensive clinical validation; substantial clinical evidence; well-established performance	Earlier-generation technology relative to newer CGM systems	Large multicenter enrollment, YSI reference measurements, standardized analytical methodology, and differences related to device generation
FreeStyle Libre 2	Bailey et al.	YSI laboratory reference	>100	14 days	~9.2	>99% Consensus Error Grid Zones A+B	Widely adopted system; extensive clinical experience; substantial evidence base	Reported performance may vary during rapid glucose excursions	YSI laboratory reference, multicenter standardized evaluation, and differences in sensor generation and study conditions

BGM, blood glucose monitoring; CGM, continuous glucose monitoring; MARD, mean absolute relative difference; YSI, Yellow Springs Instrument.

Selected contemporary CGM systems are compared according to reference methodology, sample size, wear duration, reported analytical and clinical accuracy, methodological strengths and limitations, and potential sources of methodological heterogeneity. The table is intended to provide methodological context rather than establish a direct ranking of device performance. Reported accuracy estimates should be interpreted cautiously because differences in study design, reference methods, participant characteristics, glucose distributions, pairing strategies, and analytical approaches may influence cross-study comparisons (10–14).

As shown in **Table 2**, apparent differences in MARD among contemporary CGM systems are accompanied by substantial methodological variations. Consequently, direct numerical comparisons should be interpreted cautiously, as methodological heterogeneity may contribute to observed differences in reported sensor performance (10–14).

### 3.4 Temporal pairing between CGM and BGM

The pairing strategy allowing a ±5-minute interval between CGM and fingerstick measurements represents another important methodological consideration (6). During periods of stable glucose concentration, this approach is unlikely to introduce substantial bias. However, during rapid glycemic excursions, physiological delays between blood and interstitial glucose may produce systematic disagreement that does not necessarily reflect sensor performance.

Rather than relying on a single pairing interval, future validation studies should consider sensitivity analyses using multiple matching windows (± 1, ± 2, and ±5 minutes) and stratify analyses according to glucose rate-of-change categories.

### 3.5 Lack of longitudinal performance assessment

Sensor accuracy may vary throughout the wear period because factors such as post-insertion tissue responses, sensor stabilization, enzyme degradation, and biofouling can influence analytical performance over time.

The original study reports an overall MARD but does not provide a detailed assessment of changes in sensor accuracy across the 15-day wear period (6). Such longitudinal analyses are clinically relevant because temporal variations in performance, including potential changes toward the end of sensor wear, may remain undetected when accuracy data are reported only as pooled estimates.

Mixed-effects longitudinal regression models could be considered to characterize temporal changes in analytical performance while accounting for repeated measurements within participants and sensors. Such analyses may help determine whether sensor performance changes significantly over the wear period.

### 3.6 Sensor survival bias

Approximately one-quarter of sensors did not remain functional through completion of the intended study period (6). This degree of attrition warrants further investigation because premature sensor discontinuation may not occur randomly.

If sensors that discontinue prematurely differ systematically in analytical performance from those completing the full wear period, pooled accuracy estimates may be affected by selection related to sensor survival. However, the direction and magnitude of any such bias cannot be determined without comparing performance characteristics between discontinued and successfully completed sensors.

Time-to-event methods, including Kaplan–Meier analysis and, when an adequate number of events is available, Cox proportional hazards regression, could provide additional information regarding sensor durability and factors associated with premature discontinuation.

### 3.7 Statistical uncertainty

The original study reports point estimates for several performance indicators but provides limited information regarding the statistical uncertainty surrounding these estimates (6).

Reporting confidence intervals for key accuracy outcomes, including MARD and error-grid proportions, would improve interpretation of the precision of the reported estimates. Bootstrap resampling may also provide a useful approach for quantifying uncertainty, particularly in studies with relatively small sample sizes and repeated glucose measurements.

### 3.8 Missing data mechanisms

Real-world CGM studies may generate missing observations because of sensor interruptions, participant-related factors, or technical failures. The mechanisms underlying such missingness may influence the validity of resulting accuracy estimates.

The original publication does not provide a detailed characterization of whether missing observations were considered Missing Completely at Random (MCAR), Missing at Random (MAR), or Missing Not at Random (MNAR) (6). Distinguishing among these mechanisms is methodologically important because excluding incomplete observations without considering the underlying mechanism may introduce bias.

Depending on the extent and pattern of missingness, sensitivity analyses and appropriate missing-data methods, including multiple imputation or inverse probability weighting, could be considered.

### 3.9 Limited subgroup analyses

Overall accuracy estimates may conceal clinically relevant variation across patient populations and clinical conditions.

Where sample size and study design permit, accuracy could be evaluated according to relevant characteristics such as diabetes type, age, body mass index, insulin treatment modality, HbA1c level, and glycemic variability. Sensor performance across different glucose ranges, particularly during hypoglycemia, also warrants specific evaluation because measurement accuracy in this range may have important implications for therapeutic decision-making and patient safety (9).

### 3.10 Sponsorship and analytical independence

Transparency regarding industry involvement is an important consideration in the evaluation of medical devices. The original study appropriately disclosed its funding sources and potential conflicts of interest, allowing readers to interpret the findings within this context (6).

An additional methodological consideration concerns the relationship between study sponsorship and the reference measurement system. The FreeStyle Optium blood glucose monitoring system was used as the reference method in the study (6). Where a reference method has a commercial relationship relevant to study sponsorship, transparent disclosure and independent validation can further strengthen confidence in the interpretation of diagnostic accuracy findings. Importantly, such a relationship does not in itself indicate analytical or reporting bias.

Independent validation using laboratory reference methods such as the Yellow Springs Instrument (YSI), or other appropriately validated reference systems, could provide complementary evidence regarding device performance. More broadly, independent statistical analysis, transparent access to relevant analytical data where feasible, and external replication may strengthen methodological transparency, reproducibility, and confidence in future CGM validation studies.

### 3.11 Clinical versus analytical accuracy

Analytical accuracy should not be considered synonymous with clinical performance or clinical effectiveness (15–17). Although analytical metrics quantify agreement between CGM measurements and reference glucose values, they do not fully capture the clinical implications of measurement error or how sensor performance may influence diabetes management.

Clarke and Parkes Error Grid analyses provide complementary information by evaluating the potential clinical consequences of measurement errors; however, they do not independently establish long-term effectiveness in routine diabetes care (18). A comprehensive evaluation of CGM performance should therefore integrate analytical accuracy with clinically relevant measures, including Time in Range, hypoglycemia burden, patient-reported outcomes, treatment satisfaction, and the potential impact of sensor performance on therapeutic decision-making (15–18).

Integrating these complementary dimensions may provide a more clinically meaningful assessment of emerging CGM technologies and facilitate interpretation of their performance beyond conventional analytical accuracy metrics.

## 4 Toward a Standardized Multidomain Framework for CGM Validation

The methodological considerations discussed throughout this Perspective extend beyond the evaluation of the Sinocare iCan i3 system and reflect broader challenges in the validation of contemporary continuous glucose monitoring (CGM) technologies. Despite substantial advances in sensor performance, important methodological heterogeneity persists across validation studies with respect to reference standards, glucose-pairing strategies, statistical analyses, reporting practices, and outcome measures (2, 9). As analytical differences among current-generation CGM systems continue to narrow, methodological variability may increasingly influence reported accuracy estimates and complicate direct comparisons among devices. Consequently, rigorous and standardized validation methodology is becoming increasingly important alongside continued technological innovation. The proposed six-domain framework is summarized in **Figure 1**.

**Figure 1:**
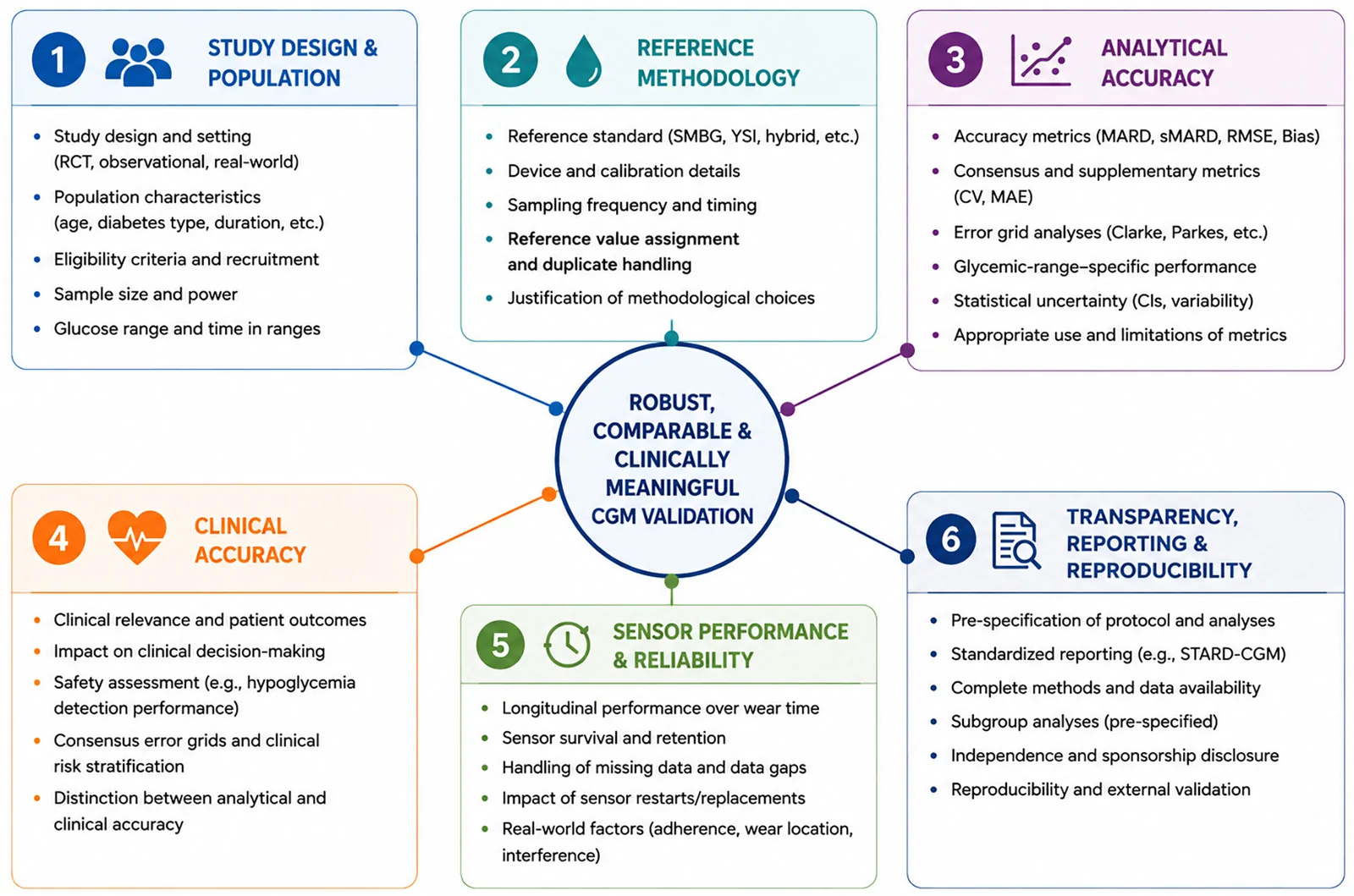
Proposed Standardized Multidomain Framework for Continuous Glucose Monitoring Validation. The proposed framework integrates six complementary methodological domains: study design and population, reference methodology, analytical accuracy, clinical accuracy, sensor performance and reliability, and transparency, reporting, and reproducibility. These interconnected domains encompass key considerations related to study design, reference standards, accuracy metrics, statistical uncertainty, clinical relevance, longitudinal sensor performance, missing data, methodological transparency, and reproducibility. Together, they provide a structured approach to enhance the methodological rigor, comparability, reliability, and clinical relevance of continuous glucose monitoring (CGM) validation studies.

The contribution of this Perspective does not lie in introducing individual accuracy metrics or validation principles, many of which have already been described (9, 19). Rather, it integrates these elements into a structured multidomain framework encompassing reference methodology, analytical and clinical accuracy, temporal pairing, longitudinal sensor performance, statistical uncertainty, missing-data assessment, subgroup analyses, sensor survival, and reporting transparency. Using the Sinocare iCan i3 study as an illustrative real-world case (6), this framework highlights how methodological choices across interconnected domains may jointly influence reported CGM performance and limit cross-study comparability. The proposed practical checklist (**Table 1**) is intended to translate these considerations into a reproducible structure for the design, analysis, and reporting of future CGM validation studies.

Whenever feasible and appropriate to the study design, laboratory reference methods such as the Yellow Springs Instrument (YSI) should be considered for analytical performance evaluation (2, 9). When real-world studies rely on capillary blood glucose measurements, investigators should comprehensively report the analytical characteristics of the reference method and consider measurement uncertainty when interpreting CGM accuracy estimates. Standardized reporting should also include complementary analytical metrics, such as MedARD, RMSE, Bland–Altman analysis, and measures of systematic bias, together with confidence intervals, longitudinal assessment of sensor performance throughout the wear period, evaluation of missing-data mechanisms, relevant subgroup analyses, and statistical approaches capable of accounting for repeated measurements and physiological variability (2, 9, 19). Clinical performance measures, including appropriate error-grid analyses, Time in Range, hypoglycemia-related outcomes, and patient-reported outcomes, should complement traditional analytical accuracy metrics to provide a more comprehensive assessment of clinical relevance (15, 16, 18, 20–22).

Equally important is greater methodological transparency through prospective protocol registration, predefined statistical analysis plans, independent statistical review where appropriate, comprehensive reporting of missing-data mechanisms, and, whenever feasible, access to anonymized paired glucose datasets. Such practices could improve reproducibility, facilitate external validation, and strengthen confidence in published findings (2, 23).

Ultimately, future advances in CGM research should encompass not only improvements in sensor technology but also greater international harmonization of validation methodology (2, 21–24). The framework proposed in this Perspective provides a practical foundation for improving comparability among emerging technologies, supporting more consistent analytical and regulatory evaluation, and promoting standardized interpretation and reporting of CGM-derived information (23–25).

By integrating analytical, clinical, longitudinal, statistical, and reporting considerations within a single structure, this Perspective contributes to ongoing efforts toward a more comprehensive and standardized approach to CGM validation.

## 5 Strengths and limitations of this perspective

This Perspective has several strengths and limitations that should be acknowledged. A key strength is the integration of analytical, clinical, longitudinal, statistical, and reporting considerations into a unified multidomain framework intended to support more standardized CGM validation. The proposed framework and practical checklist also translate methodological considerations into a structured approach that may inform the design, analysis, interpretation, and reporting of future validation studies.

Several limitations should nevertheless be considered. First, this article represents a narrative critical appraisal rather than a systematic review and therefore does not provide an exhaustive evaluation of all published CGM validation studies. Second, the methodological considerations are illustrated primarily through a single recent real-world evaluation of the Sinocare iCan i3 system (6). Although many of the issues discussed may be relevant to contemporary CGM validation more broadly, their relative importance may vary according to study design, reference methodology, participant population, and device characteristics. Finally, the proposed multidomain framework has not yet undergone formal expert consensus or prospective validation. Its recommendations should therefore be considered a contribution to ongoing methodological standardization efforts and may evolve as additional evidence and international consensus emerge.

## 6 Conclusion

The study by Bhargava et al. provides valuable real-world evidence regarding the performance of the Sinocare iCan i3 CGM system (6). Nevertheless, several methodological considerations—including the choice of reference method, interpretation of MARD alongside complementary accuracy metrics, limited characterization of statistical uncertainty, absence of detailed longitudinal performance analyses, potential survival-related bias, and incomplete characterization of missing data—should be considered when interpreting the reported findings. Moreover, direct comparisons with other contemporary CGM systems should be made cautiously because differences in study design, reference methods, participant characteristics, pairing strategies, and analytical approaches may contribute to variation in reported accuracy estimates (2, 9–14).

Future CGM validation studies would benefit from a more comprehensive and standardized framework integrating analytical and clinical accuracy, longitudinal sensor performance, appropriate statistical methodology, transparent handling of missing data, and clinically relevant outcomes (2, 9, 15–19). Greater methodological harmonization could enhance the comparability, reproducibility, and clinical relevance of CGM evaluations and ultimately support more robust evidence for clinicians, regulatory authorities, manufacturers, and people living with diabetes.

## Data Availability

The original contributions presented in the study are included in the article/Supplementary Material. Further inquiries can be directed to the corresponding author.
